# Assessing the impact of sociodemographic and lifestyle factors on oral health: a cross-sectional study in the Hungarian population

**DOI:** 10.3389/fpubh.2023.1276758

**Published:** 2023-10-20

**Authors:** Amr Sayed Ghanem, Marianna Móré, Attila Csaba Nagy

**Affiliations:** ^1^Department of Health Informatics, Institute of Health Informatics, Faculty of Health Sciences, University of Debrecen, Debrecen, Hungary; ^2^Institute of Social and Sociological Sciences, Faculty of Health Sciences, University of Debrecen, Nyíregyháza, Hungary

**Keywords:** oral health, sociodemographic, lifestyle, dental caries, periodontitis, EHIS, gingival bleeding, missing teeth

## Abstract

**Introduction:**

Oral health, a critical aspect of overall well-being, is influenced by various sociodemographic and lifestyle factors, with poor oral health associated with systemic diseases and diminished quality of life.

**Methods:**

This cross-sectional study leverages data from the Hungarian European Health Interview Survey (EHIS) representative of the Hungarian population, to conduct a comprehensive examination of the intersection between these factors and oral health, aiming to identify potential risk factors and contribute to the development of targeted interventions. The research examined associations between sociodemographic/lifestyle factors and oral health. Statistical techniques included Pearson’s Chi-square test, multivariate and ordinal logistic regression analyses. Weighting was applied to assure the representativeness of the population and enhance the validity of the survey results.

**Results:**

The study identifies gender, age, education, financial status, smoking, and self-perceived oral health as key factors influencing oral health outcomes. Notably, regular dental visits significantly reduced the risk of poor oral health and caries. Females, non-smokers, university graduates, high-income individuals, and those with good self-perceived health had fewer missing teeth and better self-perceived oral health. Teeth extractions due to decay, especially when not replaced, significantly increased the perception of poor oral health, while regular dental visits improved it.

**Discussion:**

The study highlights the need for personalized oral health interventions considering the different sociodemographic and lifestyle factors, along with promotion of healthy lifestyle, more frequent dental office visits and equitable dental care access. The findings offer the potential to inform regional oral health policies and prevention strategies, improving oral health and overall wellbeing.

## 1. Introduction

Oral health is a vital aspect of overall well-being and quality of life, influenced by a diverse array of sociodemographic and lifestyle factors (1, 2). Poor oral health has been linked to a range of adverse outcomes, including pain, functional limitations, compromised nutrition, and impaired social functioning. Moreover, emerging evidence suggests a bidirectional relationship between oral health and systemic health, with oral diseases being associated with an increased risk of cardiovascular diseases, diabetes, and adverse pregnancy outcomes (3–5).

The significance of identifying risk factors for poor oral health cannot be understated, as oral diseases can have wide-ranging implications for an individual’s overall health and quality of life. Dental caries and periodontal diseases, the most common oral health problems, can lead to pain, tooth loss, and impaired oral function, affecting one’s ability to eat, speak, and socialize. These conditions not only cause discomfort and functional limitations but can also have far-reaching consequences on systemic health (6). Emerging evidence indicates that oral health is intricately linked to systemic health, with various oral diseases being associated with an increased risk of developing serious health conditions. For example, individuals with chronic periodontal disease have been found to have a higher risk of developing cardiovascular diseases, including coronary heart disease and stroke (7).

Severe periodontal diseases and dental caries remain widespread public health challenges, affecting millions of people worldwide. The burden of severe periodontal diseases is staggering, with approximately 19% of the global adult population, accounting for over 1 billion cases, grappling with the health consequences of these conditions. These diseases not only cause significant pain and discomfort but also substantially impact individuals’ overall quality of life. Similarly, dental caries poses a significant global health concern, with an alarming 2 billion people suffering from caries of permanent teeth (8). Even more concerning is the prevalence of caries in young children, with 514 million children worldwide experiencing caries of primary teeth, potentially setting the stage for future oral health problems. The burden of oral diseases extends beyond individual suffering, as it places a substantial strain on healthcare systems and economies globally. The treatment of severe periodontal diseases and dental caries requires considerable resources, including dental care, medications, and restorative procedures, resulting in significant healthcare expenditures (9). Additionally, the impact of oral diseases on productivity, absenteeism from work or school, and reduced overall well-being further underscores the urgency of addressing these issues.

Age, as a key determinant, plays a crucial role in shaping oral health outcomes. Both young and older adult/adults individuals face distinct challenges, with children often affected by caries of primary teeth, while older adults experiencing a higher prevalence of severe periodontal diseases (10, 11). Gender disparities in oral health have also been observed, with certain conditions more prevalent in one gender over the other (12). Understanding these variations is essential for designing tailored oral health interventions.

Educational attainment is also closely linked to oral health outcomes, as individuals with lower education levels are more likely to face barriers in accessing preventive dental care and adopting healthy oral hygiene practices (13). In addition, employment status can influence oral health through factors like stress levels, access to health insurance, and the ability to seek dental services regularly (14). Financial status, a critical determinant, significantly impacts access to dental care and treatment, often leading to disparities in oral health outcomes across different sociodemographic strata (15).

When talking specifically about Hungary, a country in central eastern Europe, according to data from the World Health Organization (WHO), Hungary has the highest prevalence of dental caries in Europe (16, 17). It is alarming that a significant portion of the Hungarian population, especially those aged 65 and above (30%) and 75 and above (40%), experience complete tooth loss (18). Primary etiological factors contributing to this issue include gingivitis and periodontitis, which serve as precursors to tooth loss. One in every three individuals in Hungary suffers from gum inflammation, indicating a substantial burden of periodontal diseases among the population (19).

Furthermore, Hungary ranks the first among European countries in terms of the incidence of cancers of the oral cavity, with approximately 3,000 cases diagnosed annually. The mortality rate associated with oral cavity cancer exceeds 1,600 cases per year (20). These concerning statistics demonstrate the importance of exploring the possible risk factors that could explain exactly why the oral health status in the Hungarian population in such a shape in relation to other European is countries and provide a plausible explanation for this phenomenon.

The aim of this study is to conduct a comprehensive analysis of the association between various sociodemographic and lifestyle factors and oral health outcomes. Leveraging data from the Hungarian European Health Interview Survey, the research seeks to identify potential risk factors and protective elements that may significantly influence oral health status.

## 2. Methods

### 2.1. Study design

The data for this cross-sectional study were exclusively obtained from the 2019 Hungarian implementation of the cross-sectional European Health Interview Survey (EHIS) (21, 22). The EHIS serves to establish reliable health indicators in EU Member States and provides comprehensive information on various health aspects, including lifestyle characteristics, self-care limitations, physical activity, nutrition, health risk behaviors, and healthcare utilization and satisfaction, prevalence of several chronic conditions and also different indicators for oral health.

This study employed individualized weighting factors provided by each Member State to mitigate non-response bias and ensure the sample accurately mirrored the population structure. Adhering to Eurostat’s guidelines, the weighting process adjusted the sample’s sex and age distribution to reflect the target population (23).

The 2019 EHIS dataset used in this study was obtained from the Hungarian Central Statistical Office, which conducted and supervised the data collection and primary analysis. The dataset constitutes a representative sample of the Hungarian adult population from private households and was collected using a standardized questionnaire under the supervision of Eurostat. The rigorous stratified probability sampling approach ensures the dataset’s representativeness for the Hungarian population.

Given the focused scope of this research, only the 2019 dataset was utilized, providing a robust and up-to-date source for investigating the association between sociodemographic and lifestyle factors and oral health outcomes in Hungary.

### 2.2. Participants

The initial sample aimed to include 12,002 individuals living in private households, aged 15 and older, drawn from 510 municipalities across Hungary. However, the final dataset consists of 5,603 respondents, yielding a response rate of approximately 47%. When a selected adult individual lived in a household with a child between the ages of 6 months and 14 years, additional data on one child were collected (21).

### 2.3. Data collection

Data collection was conducted through electronic data gathering and face-to-face interviews, executed by approximately 250 enumerators. A standardized questionnaire formulated by Eurostat was employed to ensure the consistency and comparability of data across EU Member States. Participants were informed of the study’s objectives, timeline, and response options via personalized invitation letters. Responses were recorded on digital devices by the enumerators (21).

### 2.4. Variables of interest

The analysis incorporated an array of sociodemographic and lifestyle variables such as age which was segmented into three categories: 15 to 34, 35 to 64, and 65 or older. Place of residence was bifurcated into rural and urban locales. Educational attainment was categorized into three levels: less than high school, high school diploma, and university degree. Employment status was denoted as employed or unemployed.

Financial status was gaged via two measures: self-perceived financial health on a Likert scale (ranging from “good” to “bad”), and income quintiles. Lifestyle factors, such as smoking and alcohol consumption, were dichotomized into “smoker/non-smoker” and “drinker/non-drinker” respectively (24).

The study also included self-perceptions of overall and oral health, classified into “good”, “average”, and “bad”. The timing of the last dental visit was recorded as “less than six months ago”, “more than six months ago but less than a year”, and “more than a year ago”. The presence of chronic disease was noted as a binary “yes” or “no”.

Additional oral health indicators examined in this study included the presence of dental caries, gingival bleeding during brushing, tooth mobility, and the occurrence of teeth extraction due to decay. We also recorded the existence of filled teeth and prosthodontically replaced teeth, each denoted by a binary “yes” or “no”. The total count of teeth extracted due to decay (excluding wisdom teeth and those extracted for orthodontic purposes) was divided into four levels: none, 1–5, 6–19, and 20 or more. To provide a comprehensive picture of the participants’ oral health, we generated a composite variable termed “Oral Health.” This composite variable integrated the aforementioned oral health indicators: presence of dental caries, gingival bleeding during brushing, tooth mobility, and occurrence of teeth extraction due to decay. This composite variable served as the dependent variable for both univariate and multivariate analyses. To expand the scope of the analysis, various other dependent variables were utilized to examine the effects of different factors on distinct outcomes. For the bivariate analysis, dental caries was employed as an additional dependent variable.

### 2.5. Statistical analysis

Descriptive statistics were primarily presented as weighted proportions. Pearson’s Chi-square test was utilized to investigate the associations between study variables and oral health outcomes, as well as the presence of dental caries through bivariate comparisons.

To identify risk factors associated with adverse oral health outcomes, multiple logistic regression model was used. This model was focused on delineating the risks associated with poor oral health, presence of dental caries, and gingival bleeding during brushing among study participants.

An ordinal logistic regression model was employed to identify potential risk factors associated with tooth loss and self-perceived oral health status.

In order to account for population characteristics more accurately, statistical weighting was applied in Pearson’s Chi-square test and logistic regression and ordinal logistic regression analyses. However, it’s worth noting that raw numbers across different strata might appear identical (or nearly so). This occurrence of disparate percentages is a consequence of the applied statistical weighting.

In addition to the methods already outlined, a statistical significance threshold was established, whereby *p*-values less than 0.05 were considered to indicate significant findings. This cut-off was applied across all statistical analyses to determine the significance of associations between variables.

Findings from the logistic regression analyses were represented as odds ratios (ORs) and 95% confidence intervals (CIs). All statistical analyses were performed using the STATA IC Version 17.0 software package (25).

## 3. Results

The EHIS data involved a sample size of 5,603 participants. However, the number of respondents varies for specific variables due to incomplete responses. This has been accounted for in the analysis, and the respective sample size for each variable is noted in Table 1. Descriptive statistics by means of weighted proportions is presented in the same table.

**Table 1:** Sociodemographic, lifestyle, and health characteristics of people with and without active dental caries.

Variable	Category	Dental caries			
		Without dental caries (*n*, %)	With dental caries (*n*, %)	Total (*n*, %)	*p*-value
Gender	Male	1,667 (45.59)	782 (51.96)	2,449 (47.43)	**<0.001**
	Female	2,136 (54.41)	735 (48.04)	2,871 (52.57)	
Age groups	65+	1,283 (27.1)	256 (23.08)	1,539 (23.08)	**<0.001**
	15–34	805 (25.6)	419 (27.29)	1,224 (27.29)	
	35–64	1715 (47.3)	842 (49.64)	2,557 (49.64)	
Residence area	Rural	1,142 (27.59)	608 (36.86)	1750 (30.27)	**<0.001**
	Urban	2,661 (72.41)	909 (63.14)	3,570 (69.73)	
Educational levels	Lower than high school	1,577 (38.51)	868 (54.91)	2,445 (43.26)	**<0.001**
	High school	1,306 (35.31)	425 (29.06)	1731 (33.50)	
	University	920 (26.18)	224 (16.04)	1,144 (23.24)	
Employment	Unemployed	2023 (47.99)	648 (39.63)	2,671 (45.57)	**<0.001**
	Employed	1780 (52.01)	869 (60.37)	2,649 (54.43)	
Financial status	Average	2085 (54.11)	893 (59.84)	2,978 (55.77)	**<0.001**
	Good	1,246 (35.87)	358 (25.01)	1,604 (32.73)	
	Bad	394 (10.02)	238 (15.15)	632 (11.51)	
Income level	Low	650 (16.55)	469 (30.37)	1,119 (20.55)	**<0.001**
	Lower middle	815 (20.40)	321 (20.49)	1,136 (20.43)	
	Middle	806 (21.02)	274 (17.51)	1,080 (20.00)	
	Higher middle	902 (23.21)	287 (19.53)	1,189 (22.14)	
	High	630 (18.83)	166 (12.10)	796 (16.88)	
Smoking	Smoker	877 (23.37)	590 (39.80)	1,467 (28.13)	**<0.001**
	Non-smoker	2,926 (76.63)	927 (60.20)	3,853 (71.87)	
BMI	Overweight/Obese	2,245 (57.44)	928 (60.05)	3,173 (58.20)	0.102
	Normal	1,520 (42.56)	582 (39.95)	2,102 (41.80)	
Alcohol use	Drinker	2,637 (71.25)	1,042 (69.29)	3,679 (70.68)	0.178
	Non-drinker	1,166 (28.75)	475 (30.71)	1,641 (29.32)	
Chronic disease	No	1827 (52.26)	739 (52.25)	2,566 (52.25)	0.994
	Yes	1921 (47.74)	745 (47.75)	2,666 (47.75)	
Self-perceived health	Average	1,172 (27.62)	477 (29.99)	1,649 (28.30)	0.069
	Good	2,201 (62.77)	846 (59.31)	3,047 (61.77)	
	Bad	419 (9.61)	188 (10.70)	607 (9.93)	
Self-perceived oral health	Average	1,133 (27.70)	581 (38.34)	1714 (30.79)	**<0.001**
	Good	2,119 (60.13)	377 (26.57)	2,496 (50.41)	
	Bad	534 (12.16)	555 (35.08)	1,089 (18.80)	
Number of teeth extracted due to decay, not replaced	None	138 (5.39)	45 (4.01)	183 (4.01)	**<0.001**
	1 to 5	1,301 (47.74)	682 (54.77)	1983 (49.99)	
	6 to 19	730 (23.08)	465 (32.75)	1,195 (26.17)	
	More than 20	818 (23.79)	131 (8.47)	949 (18.90)	
Presence of prosthodontically replace teeth	No	1758 (50.94)	1,011 (68.59)	2,769 (56.04)	**<0.001**
	Yes	2031 (49.06)	497 (31.41)	2,528 (43.96)	
Last dental visit	More than a year ago	1986 (49.69)	959 (62.92)	2,945 (53.50)	**<0.001**
	Less than 6 months ago	1,022 (29.31)	298 (20.70)	1,320 (26.83)	
	less than a year but more than 6 months ago	759 (21.00)	240 (16.38)	999 (19.67)	

Bold values indicate statistical significance (*p* < 0.05) based on weighted Pearson’s chi-squared tests.

52.57% of the 5,320 participants (for this specific variable) were female. The majority of participants (49.84%) fell within the age range of 35 to 64 years and resided in urban areas (71.29%). Educational attainment demonstrated diversity, with 42.09%. Having education lower than high school, and employment status was nearly evenly split between employed and unemployed participants.

Financial status, largely self-assessed as average (55.90%), spanned across various income levels. The participants predominantly reported being non-smokers (71.87%) and alcohol consumers (71.26%). The high rate of overweight/obesity status at 58.20% raises potential health concerns.

Chronic disease presence was reported by 47.73% of the population, while a majority self-assessed their overall health as good (61.51%). Notwithstanding, poor oral health was prevalent, with 80.23% of participants falling into this category, despite 46.16% having visited a dentist within the last year.

Oral health issues were significant: 28.96% reported the presence of dental caries, and 66.95% had undergone tooth extraction due to decay. Conversely, gingival bleeding when brushing was relatively less reported (16.47%), suggesting this symptom is not predominant among this population.

As seen also in Table 1, dental caries showed statistically significant associations with several demographic and health factors. Gender, age, area of residence, educational level, employment status, financial status, income level, smoking status, self-perceived oral health, number of teeth extracted due to decay, and presence of prosthodontically replaced teeth all had a significant impact on dental caries prevalence, with *p*-values less than 0.001.

In contrast, factors such as BMI, alcohol consumption, presence of a chronic disease, and self-perceived health were not found to significantly correlate with dental caries prevalence, with *p*-values above the 0.05 threshold (Table 1).

Table 2 presents bivariate analysis results, revealing significant associations between overall oral health and multiple factors. Age, residential area, education level, financial status, and health-related behaviors like smoking and chronic diseases showed strong correlations with oral health (all *p* < 0.001). Self-perception of health and specific dental health indicators like tooth extractions due to decay also significantly influenced oral health (*p* < 0.001) (Table 2).

**Table 2:** Bivariate analysis of overall oral health in relation to demographic, sociodemographic, behavioral, and health-related factors.

Variable	Category	Oral Health			
		Good oral health (*n*, %)	With bad oral health (*n*, %)	Total (*n*)	*p*-value
Gender	Male	448 (19.73)	2,124 (80.27)	2,572	0.943
	Female	544 (19.81)	2,487 (80.19)	3,031	
Age groups	65+	155 (9.89)	1,473 (90.11)	1,628	**<0.001**
	15–34	491 (38.77)	785 (61.23)	1,276	
	35–64	346 (14.02)	2,353 (85.98)	2,699	
Residence area	Rural	274 (15.77)	1,540 (84.23)	1,814	**<0.001**
	Urban	718 (21.46)	3,071 (78.54)	3,789	
Educational levels	Lower than high school	335 (14.62)	2,172 (85.38)	2,507	**<0.001**
	High school	367 (21.96)	1,477 (78.04)	1,844	
	University	290 (25.68)	962 (74.32)	1,252	
Employment	Unemployed	466 (19.26)	2,331 (80.74)	2,797	0.429
	Employed	526 (20.19)	2,280 (79.81)	2,806	
Financial status	Average	459 (16.15)	2,673 (83.85)	3,132	**<0.001**
	Good	411 (27.46)	1,259 (72.54)	1,670	
	Bad	78 (12.89)	587 (87.11)	665	
Income level	Low	159 (14.41)	994 (85.59)	1,153	**<0.001**
	Lower middle	171 (16.21)	1,002 (83.79)	1,173	
	Middle	175 (17.18)	964 (82.82)	1,139	
	Higher middle	256 (22.32)	1,013 (77.68)	1,269	
	High	231 (29.67)	638 (70.33)	869	
Smoking	Smoker	233 (16.16)	1,312 (83.84)	1,545	**<0.001**
	Non-smoker	759 (21.18)	3,299 (78.82)	4,058	
BMI	Overweight/Obese	430 (14.13)	2,902 (85.87)	3,332	**<0.001**
	Normal	538 (27.23)	1,667 (72.77)	2,205	
Alcohol use	Drinker	699 (20.04)	3,207 (79.96)	3,906	0.471
	Non-drinker	293 (19.11)	1,404 (80.89)	1,697	
Chronic disease	No	659 (26.37)	2032 (73.63)	2,691	**<0.001**
	Yes	298 (12.03)	2,500 (87.97)	2,798	
Self-perceived health	Average	163 (9.81)	1,599 (90.19)	1,762	**<0.001**
	Good	776 (26.29)	2,409 (73.71)	3,185	
	Bad	39 (6.6)	584 (93.4)	623	
Self-perceived oral health	Average	141 (7.95)	1,689 (92.05)	1,830	**<0.001**
	Good	789 (32.84)	1807 (67.16)	2,596	
	Bad	44 (3.63)	1,092 (96.37)	1,136	
Number of teeth extracted due to decay, not replaced	None	51 (26.67)	143 (73.33)	194	**<0.001**
	1 to 5	65 (3.05)	1995 (96.95)	2,060	
	6 to 19	23 (2.03)	1,205 (97.97)	1,228	
	More than 20	54 (5.75)	908 (94.25)	962	
Presence of prosthodontically replace teeth	No	700 (26.84)	2,161 (73.16)	2,861	**<0.001**
	Yes	251 (9.91)	2,422 (90.09)	2,673	
Last dental visit	More than a year ago	458 (16.22)	2,650 (83.78)	3,108	**<0.001**
	Less than 6 months ago	296 (24.22)	1,071 (75.78)	1,367	
	less than a year but more than 6 months ago	206 (22.11)	839 (77.89)	1,045	
Presence of filled teeth	No	867 (26.04)	2,936 (73.96)	3,803	**<0.001**
	Yes	0 (0.0)	1,517 (100.0)	1,517	

Bold values indicate statistical significance (*p* < 0.05) based on weighted Pearson’s chi-squared tests.

Table 3 presents the results of a multiple logistic regression analysis on oral health, caries, and gum bleeding. Gender had significant disparities; females had lower odds for poor oral health (OR: 0.64, 95% CI: 0.43–0.96) and caries (OR: 0.82, 95% CI: 0.69–0.99), but higher odds for gum bleeding (OR: 1.36, 95% CI: 1.12–1.67). Age affected caries and gum bleeding, notably in the 15–34 age group, which had the highest risk. Educational attainment correlated with reduced caries, particularly for high school or university graduates. Employment was associated with better oral health (OR: 0.49, 95% CI: 0.29–0.84). Good financial status reduced the likelihood of caries (OR: 0.77, 95% CI: 0.63–0.95), and a high-income bracket showed the least odds (OR: 0.57, 95% CI: 0.41–0.79). Among lifestyle factors, non-smokers had lower odds for caries (OR: 0.78, 95% CI: 0.64–0.95).

**Table 3:** Weighted multiple logistic regression analysis of factors influencing oral health, presence of caries, and gum bleeding.

Characteristics		Oral health		Presence of caries		Gum bleeding	
		OR (95% CI)	*p*-value	OR (95% CI)	*p*-value	OR (95% CI)	*p*-value
Gender	Male						
	Female	**0.64 [0.43–0.96]**	**0.031**	**0.82 [0.69–0.99]**	**0.034**	**1.36 [1.12–1.67]**	**0.002**
Age groups	65+						
	15–34	1.21 [0.55–2.66]	0.639	**3.69 [2.6–5.24]**	**<0.001**	**5.22 [3.53–7.73]**	**<0.001**
	35–64	1.73 [0.97–3.09]	0.063	**2.02 [1.56–2.61]**	**<0.001**	**2.38 [1.77–3.2]**	**<0.001**
Residence area	Rural						
	Urban	0.99 [0.66–1.48]	0.95	0.98 [0.82–1.17]	0.789	0.95 [0.78–1.17]	0.649
Educational levels	Lower than high school						
	High school	0.75 [0.48–1.17]	0.209	**0.69 [0.56–0.85]**	**0.001**	0.88 [0.69–1.1]	0.261
	University	0.68 [0.4–1.14]	0.143	**0.64 [0.49–0.85]**	**0.002**	1.08 [0.8–1.47]	0.614
Employment	Unemployed						
	Employed	**0.49 [0.29–0.84]**	**0.009**	1.02 [0.81–1.29]	0.846	0.99 [0.78–1.25]	0.901
Financial status	Average						
	Good	1.17 [0.78–1.75]	0.441	**0.77 [0.63–0.95]**	**0.017**	0.93 [0.74–1.17]	0.569
	Bad	1.67 [0.82–3.40]	0.151	0.88 [0.67–1.14]	0.343	0.85 [0.63–1.13]	0.276
Income level	Low						
	Lower middle	1.2 [0.66–2.17]	0.556	**0.74 [0.58–0.95]**	**0.02**	0.98 [0.75–1.28]	0.862
	Middle	1.21 [0.66–2.22]	0.534	**0.68 [0.52–0.89]**	**0.005**	0.86 [0.64–1.14]	0.291
	Higher middle	1.37 [0.74–2.53]	0.317	**0.73 [0.55–0.96]**	**0.026**	0.85 [0.63–1.14]	0.279
	High	0.81 [0.44–1.5]	0.508	**0.57 [0.41–0.79]**	**0.001**	0.69 [0.47–1.01]	0.057
Smoking	Smoker						
	Non-smoker	0.95 [0.61–1.48]	0.808	**0.78 [0.64–0.95]**	**0.012**	**1.81 [1.45–2.28]**	**<0.001**
BMI	Overweight + Obese						
	Normal	0.84 [0.58–1.23]	0.375	0.89 [0.74–1.07]	0.218	0.97 [0.79–1.19]	0.747
Alcohol use	Drinker						
	Non-drinker	0.85 [0.57–1.27]	0.43	1.22 [0.99–1.49]	0.056	0.89 [0.72–1.11]	0.3
Chronic disease	No						
	Yes	1.21 [0.8–1.81]	0.37	1.18 [0.96–1.45]	0.121	1.08 [0.86–1.36]	0.519
Self-perceived health	Average						
	Good	1.15 [0.75–1.77]	0.527	1.19 [0.95–1.49]	0.125	**0.77 [0.6–0.98]**	**0.035**
	Bad	1.07 [0.54–2.15]	0.843	0.86 [0.64–1.15]	0.311	1.12 [0.83–1.51]	0.456
Self-perceived oral health	Average						
	Good	**0.5 [0.34–0.73]**	**<0.001**	**0.24 [0.2–0.3]**	**<0.001**	**0.49 [0.38–0.62]**	**<0.001**
	Bad	**2.48 [1.24–4.99]**	**0.011**	**3.25 [2.58–4.09]**	**<0.001**	**1.91 [1.5–2.41]**	**<0.001**
Number of teeth extracted due to decay, not replaced	None						
	1 to 5	**11.81 [7.16–19.48]**	**<0.001**	1.01 [0.67–1.54]	0.951	1.04 [0.64–1.69]	0.879
	6 to 19	**22.79 [11.75–44.19]**	**<0.001**	0.84 [0.53–1.32]	0.454	1.21 [0.72–2.03]	0.479
	More than 20	**13.06 [6.62–25.74]**	**<0.001**	**0.22 [0.13–0.37]**	**<0.001**	0.68 [0.38–1.22]	0.199
Presence of filled teeth	No						
	Yes	**2.78 [1.68–4.59]**	**<0.001**	**2.11 [1.68–2.67]**	**<0.001**	1.15 [0.89–1.49]	0.293
Presence of prosthodontically replace teeth	No						
	Yes	1		**0.48 [0.4–0.59]**	**<0.001**	0.88 [0.71–1.1]	0.259
Last dental visit	More than a year ago						
	Less than 6 months ago	0.77 [0.5–1.17]	0.221	**0.52 [0.42–0.65]**	**<0.001**	1.06 [0.83–1.34]	0.65
	less than a year but more than 6 months ago	1.24 [0.72–2.14]	0.441	**0.61 [0.48–0.78]**	**<0.001**	1.18 [0.92–1.53]	0.196

Bold values indicate statistical significance (*p* < 0.05). The ORs are adjusted for other variables in the model.

Self-perception of health affected gum bleeding, with ‘good’ self-perception showing lower odds (OR: 0.77, 95% CI: 0.6–0.98). Self-perceived oral health influenced all outcomes significantly, including caries (OR: 0.24, 95% CI: 0.2–0.3). Participants with more than 20 extractions had decreased odds for caries (OR: 0.22, 95% CI: 0.13–0.37). Recent dental visits lowered odds for caries (OR: 0.52, 95% CI: 0.42–0.65) but had no impact on other outcomes (Table 3).

As illustrated in Table 4, the weighted ordinal logistic regression revealed key associations. Females had higher odds of missing teeth (OR: 1.26, 95% CI: 1.09–1.45) but reported better self-perceived oral health (OR: 0.81, 95% CI: 0.7–0.93). The 15–34 age group showed markedly lower odds of missing teeth (OR: 0.1, 95% CI: 0.07–0.13). University graduates were less likely to have missing teeth (OR: 0.54, 95% CI: 0.44–0.67) and had better self-perceived oral health (OR: 0.76, 95% CI: 0.62–0.93). Financial status had a divergent effect; good financial standing was linked to better self-perceived oral health (OR: 0.73, 95% CI: 0.62–0.85). Non-smokers had both fewer missing teeth (OR: 0.72, 95% CI: 0.62–0.85) and better self-perceived oral health (OR: 0.69, 95% CI: 0.59–0.8). Individuals with more than 20 teeth extracted due to decay exhibited the most pronounced poor self-perceived oral health (OR: 7.23, 95% CI: 4.7–11.13) (Table 4).

**Table 4:** Weighted ordinal logistic regression analysis on factors influencing missing teeth (except wisdom teeth) and self-perceived oral health.

Characteristics		Missing teeth except wisdom		Self-perceived oral health	
		OR (95% CI)	*p*-value	OR (95% CI)	*p*-value
Gender	Male				
	Female	**1.26 [1.09–1.45]**	**0.002**	**0.81 [0.7–0.93]**	**0.002**
Age groups	65+				
	15–34	**0.1 [0.07–0.13]**	**<0.001**	0.92 [0.69–1.23]	0.583
	35–64	**0.4 [0.32–0.5]**	**<0.001**	1.02 [0.84–1.24]	0.85
Residence area	Rural				
	Urban	0.93 [0.8–1.07]	0.286	**0.87 [0.75–1]**	**0.047**
Educational levels	Lower than high school				
	High school	**0.77 [0.65–0.9]**	**0.002**	**0.84 [0.72–0.99]**	**0.037**
	University	**0.54 [0.44–0.67]**	**<0.001**	**0.76 [0.62–0.93]**	**0.009**
Employment	Unemployed				
	Employed	0.83 [0.69–1.01]	0.059	0.95 [0.79–1.14]	0.58
Financial status	Average				
	Good	1 [0.84–1.18]	0.963	**0.73 [0.62–0.85]**	**<0.001**
	Bad	0.96 [0.77–1.19]	0.703	**1.47 [1.21–1.79]**	**<0.001**
Income level	Low				
	Lower middle	0.98 [0.8–1.21]	0.875	**0.78 [0.64–0.95]**	**0.014**
	Middle	0.96 [0.77–1.18]	0.678	**0.79 [0.64–0.97]**	**0.024**
	Higher middle	0.89 [0.71–1.12]	0.32	0.81 [0.65–1.02]	0.073
	High	**0.63 [0.48–0.84]**	**0.002**	**0.75 [0.58–0.98]**	**0.037**
Smoking	Smoker				
	Non-smoker	**0.72 [0.62–0.85]**	**<0.001**	**0.69 [0.59–0.8]**	**<0.001**
BMI	Overweight/Obese				
	Normal	**0.76 [0.65–0.87]**	**<0.001**	1.1 [0.95–1.26]	0.198
Alcohol use	Drinker				
	Non-drinker	**1.2 [1.03–1.4]**	**0.017**	1.02 [0.87–1.18]	0.839
Chronic disease	No				
	Yes	1.08 [0.91–1.27]	0.379	0.96 [0.82–1.12]	0.605
Self-perceived health	Average				
	Good	**0.71 [0.6–0.85]**	**<0.001**	**0.56 [0.48–0.67]**	**<0.001**
	Bad	1.22 [0.98–1.52]	0.075	**1.61 [1.31–1.98]**	**<0.001**
Self-perceived oral health	Average				
	Good	**0.53 [0.45–0.63]**	**<0.001**		
	Bad	**2.72 [2.28–3.24]**	**<0.001**		
Oral health	Good				
	Bad	**4.39 [2.51–7.68]**	**<0.001**	**2.47 [1.77–3.44]**	**<0.001**
Presence of filled teeth	No				
	Yes	**0.19 [0.16–0.23]**	**<0.001**	1.13 [0.95–1.35]	0.165
Presence of prosthodontically replace teeth	No				
	Yes	**2.86 [2.43–3.36]**	**<0.001**	**0.71 [0.61–0.83]**	**<0.001**
Last dental visit	More than a year ago				
	Less than 6 months ago	**0.81 [0.68–0.97]**	**0.02**	0.85 [0.72–1.01]	0.066
	less than a year but more than 6 months ago	**0.8 [0.67–0.95]**	**0.011**	**0.66 [0.55–0.79]**	**<0.001**
Number of teeth extracted due to decay, not replaced	None				
	1 to 5			**1.53 [1.04–2.25]**	**0.031**
	6 to 19			**5.56 [3.71–8.33]**	**<0.001**
	More than 20			**7.23 [4.7–11.13]**	**<0.001**

Bold values indicate statistical significance (*p* < 0.05).

## 4. Discussion

In this study, the aim was to execute a holistic investigation of the association between a range of sociodemographic and lifestyle determinants and oral health. Distinctively employing data from the Hungarian European Health Interview Survey, a survey of national representation, this research is the first of its kind to dive into this subject matter in the context of the Hungarian population. By examining multiple factors concurrently, this study bridges a significant gap in understanding their collective impact on oral health outcomes. The findings derived from this representative sample bear considerable weight as they can be generalized to the broader Hungarian population. This marks an unprecedented step in the research of oral health in this region of Europe.

Females exhibited lower odds for poor oral health and caries but higher odds for gum bleeding. Age factored significantly, with younger participants at higher risk for caries and gum bleeding. Education level and employment were inversely correlated with caries risk. Good financial status and higher income were linked to a lower caries risk. Non-smokers showed higher likelihood for gum bleeding. Self-perceived good health led to a lower risk of gum bleeding. Positive self-perceived oral health drastically reduced odds for poor oral health, caries, and gum bleeding. The number of teeth extracted due to decay significantly correlated with poor oral health. Regular dental visits were linked to a lower caries risk.

Females had higher odds of missing teeth but better self-perceived oral health. Younger participants and those with higher education levels and income had lower odds of missing teeth and better self-perceived oral health. Non-smokers and individuals with good self-perceived health also exhibited fewer missing teeth and improved self-perceived oral health. Poor self-perception of oral health and poor oral health significantly increased the likelihood of missing teeth. Regular dental visits improved self-perceived oral health, while the number of teeth extracted due to decay significantly worsened it.

In their 2022 study, Sharon Su et al. identified notable gender disparities in oral health and related behaviors. The authors found that men typically reported worse oral health, demonstrated less optimal oral hygiene habits, and visited the dentist less frequently than women (12, 26). The current study’s results mirrored the trend of women having lower odds for poor oral health and caries, albeit with a higher predilection for gum bleeding which is in line with what Veynachter et al. (27) have concluded stating that men reported significantly lower gum bleeding prevalence than women (27). Accordingly, public health initiatives should focus on increasing men’s engagement in oral health practices while also addressing the specific oral health needs of women.

Oscarson et al. (28) associated dental caries prevalence with younger ages, rural living, lower socioeconomic status, and less frequent tooth cleaning (28). In the current research, it was similarly found that younger individuals were at higher risk for caries and gum bleeding, underscoring the ongoing need for age-specific preventive strategies.

Higher education levels are often associated with greater knowledge about and prioritization of health, including oral health. This greater awareness and knowledge could lead to better oral hygiene habits, regular dental check-ups, and a healthier diet, thus reducing the likelihood of tooth loss and caries.

As for employment, those who are employed typically have a stable income and potentially access to employee health benefits, including dental insurance. This access can make regular dental care more affordable and accessible, leading to early detection and treatment of oral health issues before they escalate to tooth loss. Furthermore, employed individuals might have a greater motivation to maintain good oral health due to professional appearances and interactions. The results of this study, indicating an inverse correlation between education level and employment with caries risk, offer robust evidence supporting the logical connection between sociodemographic factors and oral health outcomes. This resonates with the findings of numerous other research studies, affirming the connection between higher educational level, stable employment, and reduced caries risk. Consequently, these consistent patterns across different studies reinforce the credibility of these associations and underline their potential implications for oral health policies and preventive strategies (29, 30).

The study also demonstrates that those with a ‘good’ financial status had a lower chance of developing caries and had better self-perceived oral health. Higher income levels corresponded with a reduced likelihood of caries and missing teeth, and these individuals also had better self-perceived oral health. On the contrary, those with a ‘bad’ financial status reported worse self-perceived oral health. These findings indicate a clear association between financial wellbeing and oral health status. Concluding their research, Hajek et al. (31) emphasized the significant correlation between low income and poor oral health-related quality of life in the adult population (31). They pointed out the necessity of initiatives targeting the enhancement of oral health quality of life among individuals with low income. In a separate, comprehensive meta-analysis, Singh et al. (32) confirmed that a low individual or household income is linked to numerous unfavorable oral health outcomes (32). Collectively, these studies underscore the determinative role of income level and financial status on oral health, reinforcing the findings of the current study.

Examination of lifestyle factors revealed substantial impacts on dental health. Individuals who abstain from smoking not only face a diminished likelihood of developing caries but also enjoy better overall dental health and perception. This is evident in their lower propensity to suffer tooth loss and their superior self-assessment of oral health. When considering BMI, those with a weight classification within the normal range are less likely to experience tooth loss compared to those falling into overweight or obese categories. However, there was no discernible correlation between BMI and self-perceived oral health. Lastly, alcohol consumption was found to have a detrimental impact on dental health, evidenced by the elevated risk of tooth loss among individuals who consume alcohol. Research by Tezal et al. (33) discovered a moderate escalation in the severity of periodontal disease linked to alcohol use (33). Similarly, a comprehensive meta-analysis conducted by Wang et al. (34) found a clear association between alcohol consumption and an increased likelihood of periodontitis (34). These works emphasize the significance of the study’s findings, corroborating the conclusion regarding the detrimental effects of alcohol on dental health.

It is well-documented in the literature that tobacco use presents substantial risk factors for oral health. Not only is it associated with an increased prevalence of periodontitis, but it also leads to tooth discoloration. This discoloration subsequently affects the esthetic appeal of one’s smile, which could contribute to a poorer self-perception of oral health (35–38). The consensus found in these corroborating studies, underpinning the significant influence of tobacco use on oral health, lends further strength to the results in the current research. This shared understanding across multiple works not only enhances the validity of the research’s findings but also emphasizes the urgent need for interventions targeting tobacco cessation in the interest of improving oral health outcomes. Recent studies by Issrani et al. (39) and Chang et al. (40) have identified a connection between higher BMI and the presence of dental caries, periodontitis, and tooth loss, while highlighting improved oral health and frequent tooth brushing as potentially related to lower BMI (39–41). These studies echo and lend further credence to the findings of this research.

The study revealed that participants who perceived their overall health as ‘good’ were less prone to gum bleeding, although this perception did not significantly impact overall oral health or caries. Self-perceived oral health had a substantial effect on oral health outcomes: ‘good’ perception was associated with lower odds of poor oral health, caries, and gum bleeding, while a ‘bad’ perception increased the odds. The findings of the study also revealed numerous other associations. Females, urban residents, and university-educated participants reported better self-perceived oral health. Good financial status and high income level also correlated positively with better self-perceived oral health, as did non-smoking status. General health perception impacted oral health perception: those who rated their general health as ‘good’ had better oral health perception. The study also found that bad self-perceived oral health increased the likelihood of missing teeth. Individuals with bad oral health also perceived their oral health as worse. Having prosthodontically replaced teeth, recent dental visits, and fewer teeth extractions due to decay improved self-perceived oral health. Conversely, numerous extracted (and not replaced) teeth were associated with worse self-perceived oral health. Kim et al. (42) concluded that in adults aged 35–54 years, self-perceived oral health status (OHS) was closely associated with clinically determined OHS. They suggested that self-reported oral health surveys could be utilized for community oral health planning and emphasized the importance of demographic and socioeconomic variables in such studies (42). In a separate study, Costa et al. (43) found that location of residence influenced oral health. They reported worse oral health outcomes and self-perception in the rural population, indicating a higher incidence of clinical conditions linked to biofilm analysis (43). Both these studies support the findings of the current research, demonstrating consistent conclusions regarding the significance of self-perceived oral health, demographic factors, and socioeconomic variables.

### 4.1. Strengths and limitations

This study’s strengths are in its foundation on data from the Central Statistical Agency of Hungary through the European Health Interview Survey (EHIS). The robust sample size, representative of the Hungarian population, ensures that the findings hold generalizable significance. The study is notable for its broad scope within the Central Eastern European context, simultaneously considering an expansive range of sociodemographic, lifestyle, and oral health variables. The application of diverse bivariate and multivariate statistical models further bolsters the precision of the results.

However, the inherent limitations of this study must be acknowledged. Predominantly, its cross-sectional design in data collection poses constraints in discerning temporal relationships and causal inferences. Additionally, the dependence on self-reported information may have introduced recall bias or subjectivity, potentially affecting the accuracy of data collected. Despite these constraints, the study presents valuable insights that can pave the way for future research in this public health area.

## 5. Conclusion

The study affirms the strong relationships between sociodemographic, lifestyle determinants and oral health in the Hungarian population. The significant findings underscore the necessity for tailored oral health interventions that consider these determinants, particularly focusing on gender, age, socioeconomic status, lifestyle habits, and self-perceptions of health. Importantly, the need for strategies to promote healthier lifestyle choices, improved self-perception of health, and equitable access to dental care services is also highlighted. Given the study’s regional focus, it would be compelling to conduct similar research across other Central Eastern European regions to provide a broader, comparative understanding of these associations. Such investigations hold the potential to inform regional oral health policies and preventive strategies, ultimately bettering the oral health and overall wellbeing of the population.

## Data availability statement

The original contributions presented in the study are included in the article/supplementary material, further inquiries can be directed to the corresponding author.

## Ethics statement

The studies involving humans were approved by the Ethics Committee of the University of Debrecen. The studies were conducted in accordance with the local legislation and institutional requirements. Written informed consent for participation was not required from the participants or the participants’ legal guardians/next of kin in accordance with the national legislation and institutional requirements. University of Debrecen (5609-2020).

## Author contributions

AG: Conceptualization, Formal analysis, Methodology, Visualization, Writing – original draft. MM: Supervision, Writing – review & editing. AN: Supervision, Conceptualization, Project administration, Writing – review & editing.
